# Preoperative Diagnosis of Cecal Herniation Through the Foramen of Winslow

**DOI:** 10.7759/cureus.99696

**Published:** 2025-12-20

**Authors:** Tatiana Fernandez Trokhimtchouk, Álvaro Morillo Cox, Madeleine Carrión Correa, Luis F Flores

**Affiliations:** 1 General Surgery, Hospital General IESS Sur de Quito, Quito, ECU; 2 General Surgery, Pontificia Universidad Católica del Ecuador, Quito, ECU; 3 General Surgery, Universidad Internacional del Ecuador, Quito, ECU

**Keywords:** abdominal surgery, acute abdomen, bowel obstruction, cecal herniation, computed tomography, foramen of winslow hernia, internal hernia, intestinal malrotation, lesser sac

## Abstract

Foramen of Winslow hernia is an uncommon internal hernia that may present with nonspecific symptoms and be difficult to diagnose preoperatively. We present the case of a 47-year-old woman who developed sudden epigastric pain and vomiting, in whom contrast-enhanced CT demonstrated herniation of the cecum, ileocecal valve, and part of the transverse colon through the foramen of Winslow into the lesser sac, compressing the gastric antrum. Midline laparotomy revealed a mobile cecum herniated through an enlarged foramen, requiring reduction via opening of the lesser omentum; the bowel was viable, and incidental cholecystectomy and appendectomy were performed due to biliary sludge and malrotation. The patient recovered uneventfully. This case underscores the diagnostic value of CT in identifying characteristic displacement behind the hepatoduodenal ligament and reinforces the importance of prompt surgical intervention to prevent ischemia. Adding another example of this rare presentation contributes to the limited literature on cecal herniation through the foramen of Winslow.

## Introduction

Internal herniation through the foramen of Winslow is an uncommon cause of acute abdomen, representing 0.1% of all abdominal hernias and approximately 8% of internal hernias [1]. Reported cases predominantly involve adults, with most series describing presentation between the third and sixth decades of life and a slight male predominance. Fewer than 200 cases have been documented in modern literature, and diagnosis remains challenging due to nonspecific clinical presentations and the rarity of the condition [1,2].

The herniation occurs when abdominal viscera pass through the epiploic foramen into the lesser sac, posterior to the hepatoduodenal ligament. Several predisposing factors have been described, including intestinal malrotation, failure of retroperitoneal fixation producing a highly mobile cecum or ascending colon, an enlarged foramen, and increased postprandial intra-abdominal pressure [1,3]. Small bowel is the most common content, cecal herniation is particularly rare, reported only in isolated case reports and small series [3,4].

Advances in imaging have significantly improved preoperative diagnosis. While abdominal radiography and ultrasonography may demonstrate nonspecific findings such as bowel distension or free fluid, their diagnostic value is limited in this condition. Contrast-enhanced CT, typically performed in the setting of suspected acute abdomen, is now considered the modality of choice, with characteristic findings including: bowel loops located in the lesser sac, anterior displacement of the portal vein or hepatoduodenal ligament, periportal edema, and abnormal supramesocolic positioning of the cecum or ileum [1,5,6]. Early recognition is essential, as delayed intervention may lead to closed-loop obstruction, ischemia, or perforation.

Definitive management is surgical. Both laparotomy and laparoscopy have been reported, with the choice depending on bowel viability, degree of distension, and surgeon expertise. Reduction of the herniated segment, assessment of viability, and management of contributing anatomic factors constitute the core operative principles [1,3].

We report a case of cecal herniation through the foramen of Winslow associated with intestinal malrotation, diagnosed preoperatively by CT and treated successfully with open reduction. This case adds to the limited literature on cecal involvement and highlights the importance of early imaging and prompt surgical management in this rare entity.

## Case presentation

A 47-year-old woman with no significant past medical history presented to the emergency department with 7 hours of sudden-onset epigastric pain following the ingestion of a carbonated beverage. The pain was severe, constant, and associated with five episodes of vomiting. She denied fever, jaundice, bowel habit changes, or previous similar episodes.

On arrival, she was hemodynamically stable: blood pressure 128/82 mmHg, pulse 92 bpm, respiratory rate 22 rpm, temperature 36.5 °C, and oxygen saturation 94% on room air. Physical examination revealed epigastric and right upper quadrant tenderness. Her abdomen was soft and depressible, without guarding or peritoneal signs.

Initial laboratory evaluation showed mild leukocytosis (WBC 11.16 × 10^9^/L, neutrophils 9.59 × 10^9^/L) with normal hemoglobin, renal function, electrolytes, pancreatic enzymes, and liver profile. The complete results are presented in Table 1.

**Table 1:** Laboratory findings on presentation Values are reported with corresponding reference ranges. All parameters were within normal limits except for mild leukocytosis.

Parameter	Result	Reference Range
C-reactive protein (high sensitivity)	4.42 mg/L	< 5 mg/L
Prothrombin time (PT)	11.3 s	10–13 s
INR	0.95	0.8–1.2
Activated partial thromboplastin time (aPTT)	28.3 s	25–35 s
White blood cell count	11.16 ×10⁹/L	4.0–10.0 ×10⁹/L
Hemoglobin	14.4 g/dL	12–16 g/dL
Hematocrit	40.0 %	36–46 %
Lymphocytes	1.28 ×10⁹/L	1.0–3.0 ×10⁹/L
Neutrophils	9.59 ×10⁹/L	1.8–7.7 ×10⁹/L
Basophils	0.01 ×10⁹/L	0.0–0.1 ×10⁹/L
Platelets	342 ×10⁹/L	150–400 ×10⁹/L
Glucose	135.8 mg/dL	70–140 mg/dL (non-fasting)
Urea	34.19 mg/dL	10–50 mg/dL
Creatinine	0.59 mg/dL	0.6–1.1 mg/dL
Chloride	103.6 mmol/L	98–107 mmol/L
Potassium	4.36 mmol/L	3.5–5.0 mmol/L
Sodium	137.9 mmol/L	135–145 mmol/L
Total bilirubin	0.51 mg/dL	0.3–1.2 mg/dL
Direct bilirubin	0.18 mg/dL	< 0.3 mg/dL
Indirect bilirubin	0.33 mg/dL	< 1.0 mg/dL
AST (aspartate aminotransferase)	28.9 U/L	10–40 U/L
ALT (alanine aminotransferase)	27.2 U/L	7–56 U/L
Alkaline phosphatase	105 U/L	44–147 U/L
Gamma-glutamyl transferase (GGT)	22.8 U/L	9–48 U/L
Amylase	61 U/L	30–110 U/L
Lipase	30.8 U/L	0–160 U/L
Lactate	0.9 mmol/L	0.5–2.0 mmol/L

**Figure 1:**
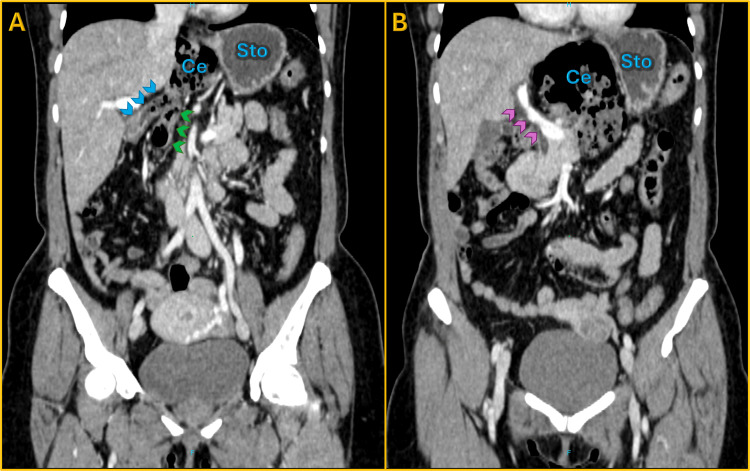
Coronal contrast-enhanced CT demonstrating cecal herniation into the lesser sac through the foramen of Winslow (A) Coronal reconstruction showing the cecum (Ce) positioned abnormally within the lesser sac, medial and posterior to the stomach (Sto). Blue arrowheads indicate the terminal ileum entering the lesser sac, and green arrowheads highlight the transverse colon. (B) Coronal reconstruction depicting the main portal vein (purple arrowheads) displaced anteriorly by the herniated bowel. The cecum (Ce) in the lesser sac, medial to the stomach (Sto), illustrates the characteristic posterior trajectory of the herniating segment behind the hepatoduodenal ligament.

**Figure 2:**
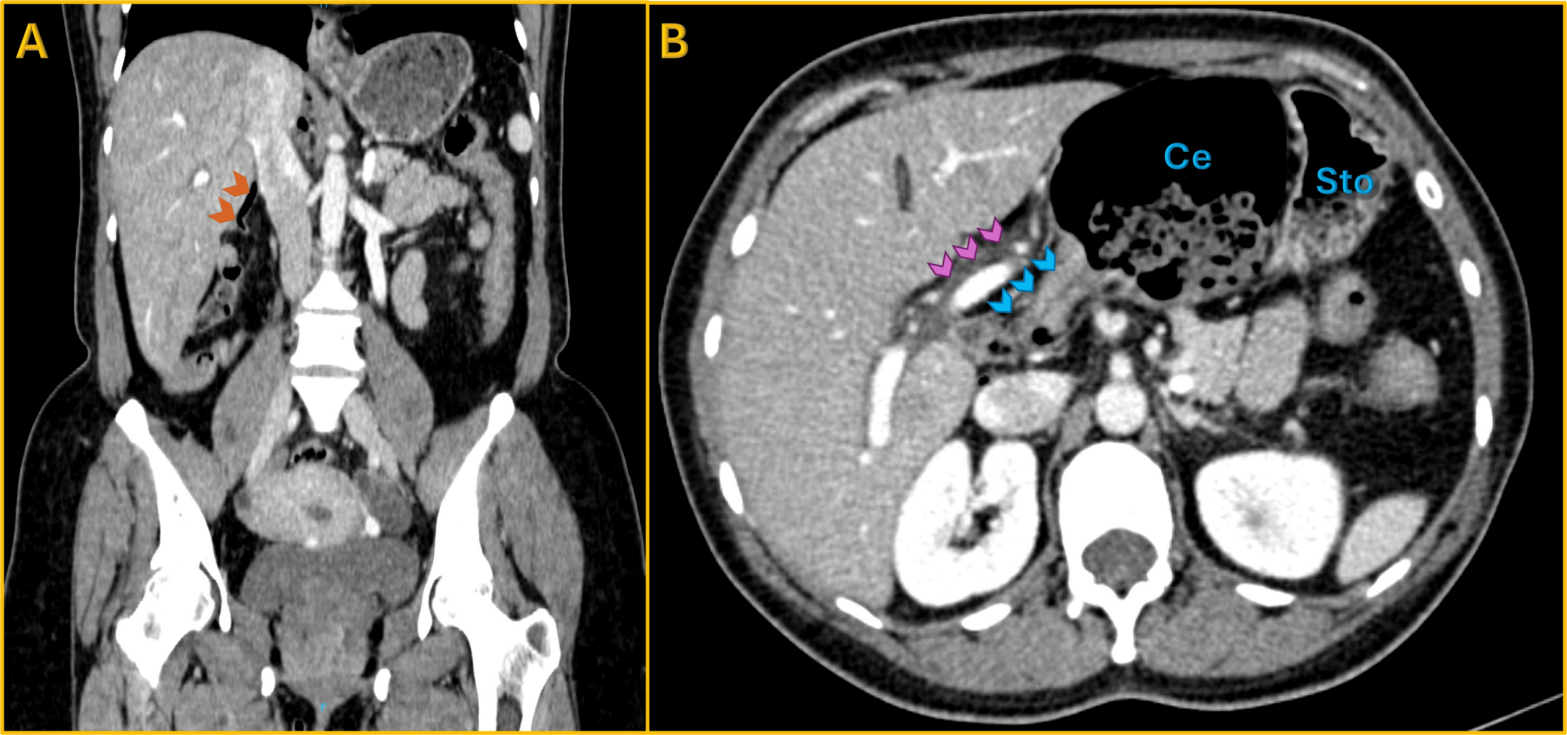
Subhepatic appendix and axial confirmation of cecal herniation behind the portal vein (A) Coronal contrast-enhanced CT showing the appendix in an abnormal subhepatic position (orange arrowheads). (B) Axial CT image demonstrating the main portal vein (purple arrowheads) displaced anteriorly, with the terminal ileum coursing behind it into the lesser sac (blue arrowheads). The cecum (Ce) is visualized within the lesser sac, medial to the stomach (Sto), confirming the path of herniation through the foramen of Winslow.

She was initially suspected to have biliary colic, but the severity and persistence of her pain prompted the emergency physicians to obtain cross-sectional imaging. A first non-contrast CT scan, obtained as part of the initial evaluation per hospital protocol, demonstrated a markedly dilated cecum in an abnormal supramesocolic position, raising concern for cecal volvulus versus a rotational anomaly of the right colon. General surgery was consulted, and a contrast-enhanced CT was requested for further evaluation. This subsequent study revealed herniation of the cecum, ileocecal valve, and proximal transverse colon through the foramen of Winslow into the lesser sac, positioned posterior to the stomach and compressing the gastric antrum, with associated periportal edema but no signs of ischemia. The superior mesenteric artery and vein demonstrated a normal anatomic relationship. Together, these findings confirmed the diagnosis of internal hernia through the foramen of Winslow (Figures 1-2).

Given the diagnosis of an internal hernia with evolving obstruction, the patient was taken for emergent laparotomy. A supra- and infraumbilical midline incision was performed. Intraoperatively, the cecum, ileocecal valve, and part of the transverse colon were found to be herniated through a markedly widened foramen of Winslow, measuring approximately 7 cm in diameter. The herniated viscera were significantly distended and adhered to the hernia sac, which extended posterior to the stomach into the lesser sac and contained inflammatory fluid. The right colon demonstrated a non-retroperitonealized, abnormally mobile configuration consistent with a rotation anomaly. The structures of the porta hepatis were intact, and a Riedel’s lobe of the liver was noted.

Reduction could not be achieved through simple traction. The lesser omentum was opened to access the lesser sac, allowing careful traction-countertraction maneuvers that ultimately achieved full reduction of the herniated bowel. After reduction, the colon and small bowel were inspected and found to be completely viable, with no ischemia.

Given the presence of biliary sludge in a thin-walled gallbladder and the extensive manipulation required around the hepatoduodenal ligament during reduction, the surgical team proceeded with an incidental cholecystectomy. In the context of a rotation anomaly characterized by colonic nonfixation, an incidental appendectomy was also performed to prevent future diagnostic uncertainty. A closed-suction Jackson-Pratt drain was placed through the foramen into the lesser sac.

The estimated blood loss was 150 mL. No intraoperative complications occurred.

Postoperatively, the patient had an uneventful recovery, with adequate pain control, early mobilization, progressing diet, and no inflammatory response. The nasogastric and urinary catheters were removed on postoperative day 1. She was discharged home on postoperative day 2 in stable condition, tolerating an oral diet and without gastrointestinal symptoms, after removal of the drain. At outpatient follow-up one week later, she remained asymptomatic and was recovering well.

## Discussion

Herniation through the foramen of Winslow is a rare cause of acute abdomen and often presents a diagnostic challenge due to its nonspecific clinical features and variable anatomical substrates. Although historically diagnosed intraoperatively, modern imaging has enabled earlier recognition, with contrast-enhanced CT now regarded as the preferred diagnostic modality because it reliably demonstrates hallmark findings such as bowel loops positioned in the lesser sac, anterior displacement of the hepatoduodenal ligament or portal vein, periportal edema, and abnormal supramesocolic positioning of the cecum or ileum [1,5-7]. Our case demonstrated these characteristic radiologic signs, including a retroportal trajectory of the ileocecal segment and compression of the gastric antrum, confirming that the preoperative diagnosis was consistent with the evolving diagnostic capabilities described in contemporary literature.

Predisposing factors described across multiple series include excessive mobility of the right colon or cecum due to deficient retroperitoneal fixation, intestinal malrotation, elongation of the mesentery, a congenitally wide or distensible foramen of Winslow, and sudden increases in intra-abdominal pressure such as postprandial gastric distension [1,3,4]. Cecal herniation represents an uncommon variant, appearing only in isolated reports and small case series, and is frequently associated with malrotation or persistent ascending mesocolon, features present in our patient and repeatedly identified as substrates favoring right-sided visceral displacement through the foramen [2,3,8]. Because the clinical presentation may mimic biliary colic, gastroduodenal disease, or even cecal volvulus, depending on the position of the cecum and appendix, diagnostic confusion is common, and initial suspicion of a biliary etiology in our case reflects a pattern well documented in recent reviews [5,6].

Surgical management remains the cornerstone of treatment due to the risk of closed-loop obstruction, ischemia, and perforation. Both laparoscopic and open approaches have been reported, with laparoscopy increasingly used in cases without severe bowel distension or when reduction is technically feasible [3,7]. However, laparotomy continues to be preferred when the bowel is markedly distended or when anatomical distortion impairs safe manipulation, as was the case here. Reduction often requires opening the lesser omentum to access the lesser sac when simple traction is unsuccessful, a technique commonly described in the literature and consistent with our operative experience. After reduction, assessment of bowel viability is essential, and most cases diagnosed early, similar to this one, avoid the need for resection, reflecting favorable outcomes reported in recent series [5,6].

The management of associated findings, such as a subhepatic appendix or gallbladder pathology, varies. Some authors describe performing appendectomy in the presence of malrotation to prevent future diagnostic uncertainty, while others justify cholecystectomy when gallbladder disease is present or when manipulation of the hepatoduodenal structures may complicate future access [3,4]. In our case, an incidental cholecystectomy was performed due to biliary sludge and the extensive dissection around the hepatoduodenal ligament, and an incidental appendectomy was performed, given the malrotated anatomy. Closure of the foramen remains controversial; most authors recommend leaving the foramen unclosed to avoid injury to the portal triad, unless a markedly enlarged defect or recurrent risk is identified. Alternative strategies described in isolated reports include cecopexy or fixation of a mobile right colon when excessive mobility is considered contributory [1,4,6,8]. In accordance with prevailing consensus and given the absence of recurrent risk factors after reduction, the foramen was not sutured in our patient.

Postoperative outcomes are generally excellent when early diagnosis and timely surgery prevent bowel ischemia. Our patient’s rapid recovery and absence of complications align with the favorable postoperative courses described in recent reports, underscoring the value of prompt imaging, early operative intervention, and careful intraoperative assessment [5-7]. Overall, this case illustrates several key elements emphasized across the contemporary literature: the diagnostic difficulty and variable presentation of foramen of Winslow hernia, the importance of recognizing predisposing anatomic factors such as malrotation, the central role of CT in establishing diagnosis, the necessity for prompt surgical reduction, and the generally favorable outcomes when bowel viability is preserved. By adding a well-documented example of cecal herniation associated with malrotation, this report contributes to the expanding body of evidence characterizing this uncommon and often underrecognized entity.

## Conclusions

Foramen of Winslow hernia remains a rare and often deceptive cause of acute abdomen, with cecal involvement representing an uncommon variant typically associated with intestinal malrotation or excessive right colonic mobility. Early recognition is essential, and contrast-enhanced CT has become the cornerstone of diagnosis by demonstrating characteristic displacement patterns within the lesser sac. Prompt surgical intervention is required to prevent ischemic complications, and most patients, particularly those diagnosed before vascular compromise, can be managed with reduction alone. Our case illustrates the importance of maintaining suspicion for internal herniation in patients with atypical supramesocolic cecal positioning, highlights the anatomic substrates that facilitate this entity, and reinforces the effectiveness of timely operative management. By contributing another example of cecal herniation associated with malrotation, this report adds to the limited but growing literature on this rare presentation and underscores the value of careful imaging interpretation and decisive surgical treatment.
